# Large-Diameter Diaphragm Fabry–Pérot Interferometer for High-Sensitivity Temperature Sensing Using a Hermetically Sealed Tunable Medium: Up to 190 nm/K

**DOI:** 10.3390/s26134071

**Published:** 2026-06-26

**Authors:** Anthony Weir, Dubhaltach Mac Lochlainn, Helio Musselwhite-Veitch, Gerard Dooly, Dinesh Babu Duraibabu

**Affiliations:** 1 Center for Robotics and Intelligent Systems (CRIS), Department of Electronic and Computer Engineering, University of Limerick, V94 T9PX Limerick, Ireland; anthony.weir@ul.ie (A.W.); musselwhiteveitch.helio@ul.ie (H.M.-V.); gerard.dooly@ul.ie (G.D.); 2 School of Engineering, University of Limerick, V94 T9PX Limerick, Ireland; 3 NSAI National Metrology Laboratory, Griffith Avenue Ext., Glasnevin, D11 E527 Dublin, Ireland; dubhaltach.maclochlainn@nsai.ie; 4 Department of Mechatronic Engineering, School of Engineering and Design, Atlantic Technological University, Ash Ln, Ballytivnan, F91 YW50 Sligo, Ireland

**Keywords:** optical fibre sensor, extrinsic Fabry–Pérot interferometry, EFPI, diaphragm, temperature sensor, hermetically sealed cavity, tuneable sensitivity, krytox GPL-105

## Abstract

This paper presents a proof-of-concept investigation into a novel hermetically sealed tunable-medium Extrinsic Fabry–Pérot Interferometer (EFPI) temperature sensor architecture. A series of tuneable-sensitivity EFPI temperature sensors is demonstrated, comprising a large-diameter fused silica diaphragm with a 800 μm diameter, significantly exceeding conventional designs (typically ∼125 μm), with polished diaphragm thicknesses ranging from 28 to 49 μm, housed in hermetically sealed rigid melting point capillaries with a 1.8 mm internal diameter. By exploiting thermally induced pressure differentials generated by a tunable Krytox GPL 105 oil/air fill fraction within the sealed rigid cavity, the sensors demonstrate a continuously tuneable sensitivity design space spanning 0.45 to 190 nm/K. An exact nonlinear thermal pressure model is derived and validated, replacing the linearised approximation which is shown to be inapplicable at fill fractions approaching unity. The low-sensitivity configuration (0.45 nm/K) was characterised at the National Standards Authority of Ireland (NSAI) National Metrology Laboratory against ITS-90 fixed points: the Triple Point of Water (273.16 K) and the Gallium Fixed Point (302.9146 K), with traceability to the International Temperature Scale of 1990 (ITS-90), yielding an instrument-limited resolution of <1.1 mK, consistent with the metrological validation environment. The high-sensitivity configurations (21 and 190 nm/K) were characterised on a laboratory bench, achieving instrument-limited theoretical resolutions of <24 μK and <2.6 μK respectively, pending future metrological validation. The 190 nm/K sensitivity represents an improvement of approximately 21.7× over the closest directly comparable prior Citationutilised fusion splicing and manual polishing. Future development priorities include metrological validation of the high-sensitivity configurations, long-term stability characterisation, thermal cycling, and progression towards an all-glass hermetically sealed construction.

## 1. Introduction

The ability to monitor minute temperature variations at sub-degree level is of significant interest across a range of fields including industrial process control, biomedical monitoring, precision manufacturing, and environmental sensing. In environments where electromagnetic interference or harsh industrial conditions are present, and where miniaturisation is a requirement, conventional electrical temperature sensors are constrained. Optical fibre sensors offer inherent immunity to electromagnetic interference, capacity for remote sensing, and compact geometry, making them well-suited to such environments.

In the biomedical field, fibre optic sensors have gained recognition in applications such as laser ablation monitoring, where localised temperature changes must be measured accurately to control tissue destruction, motivate the development of high-sensitivity, miniaturised optical temperature sensors capable of resolving sub-degree temperature changes.

Extrinsic Fabry–Pérot-Interferometry (EFPI) exploits the principles of light interference within a precisely controlled cavity, where deflection of a diaphragm modulates the optical signal. First introduced in 1897 by Fabry and Pérot [1], this interferometric technique has been widely applied to sensing applications. The practical fabrication of EFPI sensors is challenging, and a fundamental trade-off exists between sensitivity and mechanical robustness (Li et al. [2]). Several diaphragm materials and construction approaches have been investigated, including epoxy adhesive films (Zhang et al. [3]), polymeric diaphragms Eom et al. ([4]), and nanoscale diaphragms as thin as 170 nm (Liu et al. [5]). The integration of material properties, design parameters, and fabrication methods plays a fundamental role in determining sensor performance in terms of sensitivity, durability, and measurement range (Mishra [6]).

In the context of temperature sensing, conventional fibre Bragg grating (FBG) approaches demonstrate sensitivities of approximately 0.01 nm/K, whilst other fibre optic temperature sensors have demonstrated sensitivities in the range 2 to 6.6 nm/K (Qian et al. [7]; Xu et al. [8]). EFPI-based temperature sensors have been demonstrated by McGuinness et al. [9] using a polyvinyl alcohol diaphragm of 8 μm thickness and 132 μm diameter. The closest directly comparable prior art to the present work is that of Poeggel et al. [10], who demonstrated an oil-filled cavity EFPI temperature sensor with a sensitivity of 8.77 nm/K using a diaphragm of approximately 130 μm in diameter, operating on the same thermal–pressure–mechanical transduction principle as the present work.

This paper presents a proof-of-concept investigation into a novel hermetically sealed tunable-medium EFPI temperature sensor architecture. The two key features of the design are: a significantly larger diaphragm diameter of 800 μm, substantially exceeding conventional designs (typically ∼125 μm), which enhances the mechanical sensitivity of the EFPI; and a hermetically sealed rigid housing containing a tunable Krytox GPL 105 oil/air fill fraction, which acts as a thermal–pressure–mechanical conversion stage. By varying the oil/air ratio at fabrication, the sensor sensitivity can be continuously tuned across more than two orders of magnitude, from 0.45 to 190 nm/K, representing an improvement of approximately 21.7× over the closest comparable prior art [10]. An exact nonlinear thermal pressure model is derived and validated, replacing a linearised approximation shown to be inapplicable at fill fractions approaching unity. Metrological validation against ITS-90 fixed points was conducted at the National Standards Authority of Ireland (NSAI) National Metrology Laboratory for the low-sensitivity configuration, with the high-sensitivity configurations bench-characterised as preliminary demonstrations of the upper end of the tuneable design space. Poeggel et al. [10] reported their findings as a conference paper, whilst the only other directly comparable diaphragm-based EFPI temperature sensor study, McGuinness et al. [9], presented a qualitative analysis without a quantified sensitivity figure. To the authors’ knowledge, the present work therefore represents the first peer-reviewed journal publication reporting a quantified sensitivity for a diaphragm-based EFPI temperature sensor employing thermal pressure and mechanical transduction.

## 2. Literature

Fabry–Perot interferometry was first introduced in 1897 (Fabry and Pérot [1]) and has been used extensively within the role of sensing (Rao [11]). FPI sensors have found application across a broad range of fields. Poeggel et al. [12] constructed an EFPI with an integrated FBG. It consisted of an SMF 10/125 μm, a 130/200 μm capillary spliced to a 200 mm Outer Diameter (OD) multi-mode fibre which is in turn spliced to the SMF ensuring that there is an air gap of 20–30 μm between the tip of the SMF and the inside face of the multimode fibre. The diaphragm was reduced to 2 μm through polishing and HF etching. The FBG is achieved using UV exposure and phase mask technique. The FBG was spliced to the cavity 0.5 mm from the fibre tip. Liquid sealing or encapsulating has been readily demonstrated in temperature sensing.

McGuinness et al. [9] demonstrated an EFPI-based temperature sensor which consisted of an SMF spliced to a quartz capillary. The diaphragm was manufactured by immersing it in Polyvinyl alcohol (PVA). The completed EFPI was then sealed in a fluid-filled housing. The novel sensor and an all-glass EFPI were then placed in a thermogravimetric analyser (TGA) for comparison, with the novel sensor demonstrating greater sensitivity than the all-glass EFPI.

Zheng et al. [13] demonstrated a gel-filled capillary EFPI by splicing a single mode fibre (SMF) to a hollow core fibre (HCF) and filling and curing the gel within the cavity. The sensor was tested over a temperature range of 30 to 60 °C and demonstrated sensitivities of 0.999 to 1.174 nm/K. Chen et al. [14] demonstrated a fibre optic temperature sensor using a polydimethylsiloxane (PDMS)-filled air-microbubble Fabry–Pérot interferometer in a hollow core fibre, achieving a sensitivity of 2.703 nm/K over a range of 19.3 K. Liu et al. [15] demonstrated a large-cavity EFPI with an integrated FBG and a UV-cured adhesive end face, in which the thermal expansion of the adhesive diaphragm modifies the free spectral range of the EFPI, achieving a sensitivity of 4.665 nm/K over a 35 K range with a diaphragm diameter of 150 μm. Zheng et al. [13] demonstrated a UV glue spliced EFPI temperature sensor achieving a sensitivity of 1.174 nm/K over a 30 K range with a diaphragm diameter of 75 μm.

Several fibre Bragg grating (FBG)-based temperature sensors have also been reported in the literature. Samset et al. [16] demonstrated temperature monitoring during magnetic resonance imaging (MRI) procedures using a system comprising 10 fibre Bragg Gratings (FBGs) on a single mode fibre. Yan et al. [17] demonstrated a strain-decoupled sensitised FBG achieving a sensitivity of 0.040 nm/K over a 40 K range. Wang et al. [18] demonstrated an FBG sensor fabricated using chemical gilding and electroplating, achieving a sensitivity of 0.024 nm/K over a 95 K range. Khan et al. [19] demonstrated an FBG-based temperature sensor achieving a sensitivity of 0.013 nm/K over a 30.5 K range with a resolution of 120 mK. Poeggel et al. [20] demonstrated a combined EFPI pressure and temperature sensor with an integrated FBG, in which the FBG element provides temperature sensitivity of 0.011 nm/K over a 100 K range with a diaphragm diameter of 60 μm. In this device the diaphragm functions as the pressure sensing element whilst the FBG provides the temperature response.

The closest directly comparable prior art to the present work is that of Poeggel et al. [10], who demonstrated a high-resolution optical-fibre temperature sensor based on an EFPI surrounded by an oil-filled outer cavity. The thermal expansion of the oil compresses the air within the EFPI cavity, driving diaphragm deflection and producing a temperature-dependent spectral shift, following the same thermal–pressure–mechanical transduction principle employed in the present work. A sensitivity of 8.77 nm/K was reported over a 7 K range using a diaphragm diameter of approximately 130 μm. The present work extends this approach through the use of a significantly larger diaphragm diameter of 800 μm, a hermetically sealed rigid housing with a tunable Krytox GPL 105 oil/air fill fraction, and an exact nonlinear thermal pressure model, achieving sensitivities of up to 190 nm/K, representing an improvement of approximately 21.7× over [10].

### Comparative Sensor Tables

Table 1 presents a broader contextual overview of fibre optic temperature sensing approaches. Table 2 presents a like-for-like comparison restricted to diaphragm-based EFPI temperature sensors, which represents the appropriate comparator class for the present work.

## 3. Materials and Methods

The following section is divided into three subsections, detailing sensor theory, hermetically sealed oil and air-filled cavity which encapsulates the manufactured EFPI sensor [21].

### 3.1. Sensor Theory

The high-resolution temperature sensor is based on an EFPI encapsulated in an outer capillary, which is filled with a mixture of Krytox GPL 105 oil [22] and air.

**Figure 1:**
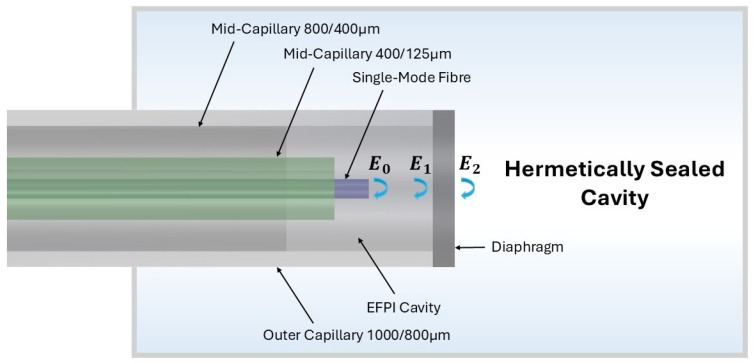
Schematic diagram of the hermetically sealed cavity EFPI sensor.

The light travels along the single mode fibre (SMF) to the Fabry–Pérot-Interferometer (FPI) sensing element $E_{0}$. Then another portion of the light is reflected at the inner face of the diaphragm $E_{1}$, whilst the remainder of the light $E_{2}$ is reflected at the outer face of the diaphragm as shown in Figure 1 and as defined in Equation (1).

(1) $$I={\left(\vec{E_{0}}+\vec{E_{1}}+\vec{E_{2}}\right)}^{2}$$

The sensor cavity is filled with air with a refractive index $n_{0}$ and a length $L_{c}$. The diaphragm thickness is $L_{d}$ with a refractive index $n_{1}$. The reflected light from the interfaces (with a wavelength λ) creates an interference pattern in the form of an FPI spectrum. The FPI sensing element measures the deflection of the diaphragm, i.e., the change in the sensor cavity length by analysing changes in the FPI spectrum as defined in Equation (2) [23].

(2) $$I\left(\lambda \right)=E_{0}\;\cdot \;E_{1}\;\cdot \;\cos\left(\frac{4\pi n_{0}L_{c}}{\lambda }\right)+E_{0}\;\cdot \;E_{2}\;\cdot \;\cos\left(\frac{4\pi (n_{0}L_{c}+n_{l}L_{d})}{\lambda }\right)+E_{1}\;\cdot \;E_{2}\;\cdot \;\cos\left(\frac{4\pi L_{d}n_{1}}{\lambda }\right)$$

#### 3.1.1. Plate Mechanics

The SI units for the calculations below are shown in Table 3 [24]. The mechanical characteristics of the diaphragm can be calculated from the following equations, where

The Flexural Rigidity is given as ([24], p. 457) as defined in Equation (3)

(3) $$D=\frac{E\;\cdot \;h^{3}}{12(1-\nu^{2})}$$

The deflection is given as ([24], p. 488) as defined in Equation (4)

(4) $$y_{\max}=\frac{q\;\cdot \;a^{4}}{64\;\cdot \;D}$$

Sensitivity is defined as deflection per unit pressure and therefore

(5) $$S=\frac{y_{\max}}{q}$$

Substituting Equation (4) for $y_{\max}$ we get

(6) $$S=\frac{3\left(1-\nu^{2}\right)\;\cdot \;a^{4}}{16\;\cdot \;E\;\cdot \;h^{3}}$$

#### 3.1.2. Thermal Pressure

For any change in temperature ΔT inside the gas/fluid-filled rigid encapsulation, an induced thermal pressure will result. For the aforementioned rigid cavity, this relationship is governed by Gay-Lussac’s Law, which states that at constant volume the pressure of a gas is directly proportional to its absolute temperature [25,26]:

(7) $$\frac{P}{T}=\mathrm{constant}$$

At the initial state $\left(P_{0},T_{0}\right)$ and a new state $\left(P_{1},T_{1}\right)$:

(8) $$\frac{P_{0}}{T_{0}}=\frac{P_{1}}{T_{1}}$$

(9) $$P_{1}=P_{0}\;\cdot \;\frac{T_{1}}{T_{0}}$$

The change in pressure ΔP is therefore:

(10) $$\Delta P=P_{1}-P_{0}=P_{0}\;\cdot \;\frac{T_{1}}{T_{0}}-P_{0}=P_{0}\left(\frac{T_{1}-T_{0}}{T_{0}}\right)$$

Substituting $P_{0}\;\cdot \;T_{1}/T_{0}$ for $P_{1}$ and replacing $T_{1}-T_{0}$ with ΔT:

(11) $$\Delta P_{\mathrm{gas}}=P_{0}\left(\frac{\Delta T}{T_{0}}\right)$$

where $P_{0}$ is the initial pressure in the sensor, $T_{0}$ is the initial absolute temperature in Kelvin, and ΔT is the change in temperature.

To enhance the thermally induced pressure response, the encapsulation cavity is partially filled with a thermally expansive fluid, with the remaining volume occupied by air. The fluid chosen is Krytox GPL 105 oil [22], which exhibits a volumetric thermal expansion coefficient of $\alpha_{f}\approx 1.09\times 10^{-3}$ K^−1^. As the oil expands within the sealed rigid cavity, it compresses the remaining air into a progressively smaller space, generating a pressure increment over and above the baseline gas response.

Let $V_{c}$ denote the total cavity volume, $V_{f}$ the volume occupied by fluid, and $V_{a}=V_{c}-V_{f}$ the volume of air remaining in the cavity, such that the oil volume fraction is defined as $\phi =V_{f}/V_{c}$, where:

(12) $$V_{a}=\left(1-\phi \right)V_{c}$$

For a temperature rise ΔT, the volumetric expansion of the fluid is:

(13) $$\delta V_{f}=\alpha_{f}\;V_{f}\;\Delta T=\alpha_{f}\;\phi \;V_{c}\;\Delta T$$

Since the cavity is rigid, the expanding fluid compresses the air volume $V_{a}$ isothermally. Before heating, the air occupies volume $V_{a}$ at pressure $P_{0}$:

(14) $$P_{0}\;\cdot \;V_{a}=\mathrm{constant}$$

After heating, the oil expansion $\delta V_{f}$ reduces the available air volume to $V_{a}-\delta V_{f}$ at a new higher pressure $P_{1}$. Applying Boyle’s Law (PV=constant at constant temperature) between the two states:

(15) $$P_{0}\;V_{a}=P_{1}\;\left(V_{a}-\delta V_{f}\right)$$

Solving for $P_{1}$:

(16) $$P_{1}=P_{0}\;\cdot \;\frac{V_{a}}{V_{a}-\delta V_{f}}$$

The pressure increment $\Delta P_{\mathrm{fluid}}=P_{1}-P_{0}$ is the additional pressure generated by the oil expansion:

(17) $$\Delta P_{\mathrm{fluid}}=P_{0}\;\cdot \;\frac{V_{a}}{V_{a}-\delta V_{f}}-P_{0}=P_{0}\;\cdot \;\frac{\delta V_{f}}{V_{a}-\delta V_{f}}$$

Substituting $\delta V_{f}=\alpha_{f}\phi V_{c}\Delta T$ and $V_{a}=\left(1-\phi \right)V_{c}$, with $V_{c}$ cancelling yields the exact expression for the fluid displacement pressure increment:

(18) $$\Delta P_{\mathrm{fluid},\mathrm{exact}}=P_{0}\;\cdot \;\frac{\alpha_{f}\phi \Delta T}{\left(1-\phi \right)-\alpha_{f}\phi \Delta T}$$

For modest fill fractions where $\alpha_{f}\phi \Delta T\ll \left(1-\phi \right)$, the denominator may be approximated as (1−ϕ) alone, yielding the linearised expression:

(19) $$\Delta P_{\mathrm{fluid},\mathrm{linear}}\approx P_{0}\;\cdot \;\frac{\alpha_{f}\phi }{1-\phi }\;\cdot \;\Delta T$$

This linearised approximation is valid where the oil expansion per kelvin is small, relative to the remaining air volume. At fill fractions approaching unity this condition is violated and the exact expression (Equation (18)) must be used. The exact expression is only defined for fill fractions below the physical ceiling:

(20) $$\phi <\frac{1}{1+\alpha_{f}\Delta T}=\frac{1}{1+1.09\times 10^{-3}}=99.891\%$$

This ceiling is a thermodynamic property of the fill fluid alone, depending only on $\alpha_{f}$ and the temperature step ΔT, and is independent of diaphragm geometry. It represents the fill fraction at which the oil expansion per kelvin exactly equals the remaining air volume, leaving no air space to compress. No sensor using Krytox GPL-105 can function over a 1 K range at or above this fill fraction, regardless of diaphragm design.

The total thermally induced pressure in the composite cavity is the sum of the gas and fluid contributions $\Delta P_{\mathrm{total}}=\Delta P_{\mathrm{gas}}+\Delta P_{\mathrm{fluid}}$:

(21) $$\Delta P_{\mathrm{total}}=P_{0}\left(\frac{\Delta T}{T_{0}}\right)+P_{0}\;\cdot \;\frac{\alpha_{f}\phi \Delta T}{\left(1-\phi \right)-\alpha_{f}\phi \Delta T}$$

For the ϕ=0.80 sensor where the linearised approximation is valid, this reduces to:

(22) $$\Delta P_{\mathrm{total}}\approx P_{0}\;\Delta T\left(\frac{1}{T_{0}}+\frac{\alpha_{f}\;\phi }{1-\phi }\right)\;\left(\phi \leq 0.90\right)$$

It follows from Equation (18) that the thermally induced pressure increases with oil fill fraction ϕ. As ϕ increases, a greater proportion of the cavity is occupied by fluid, leaving a smaller air volume to absorb the thermal expansion. The same volumetric displacement of fluid is therefore compressed into a progressively smaller air space, producing a disproportionately larger pressure rise per degree of temperature change. Consequently, a higher oil-to-air ratio results in a greater thermally induced pressure differential across the diaphragm, which in turn drives greater deflection for a given temperature change. Since diaphragm deflection is the measurable output of the EFPI sensor, the oil fraction ϕ provides a direct and controllable means of tuning the sensor’s thermal sensitivity at fabrication, without any modification to the diaphragm geometry or material. This thermally induced pressure differential acts between the composite encapsulation and the EFPI air cavity, deflecting the sensor diaphragm and producing a measurable change in cavity length, as defined in Equation (2) (Duraibabu et al. [23]).

### 3.2. Encapsulating the Sensor in a Hermetically Sealed Housing

**Figure 2:**
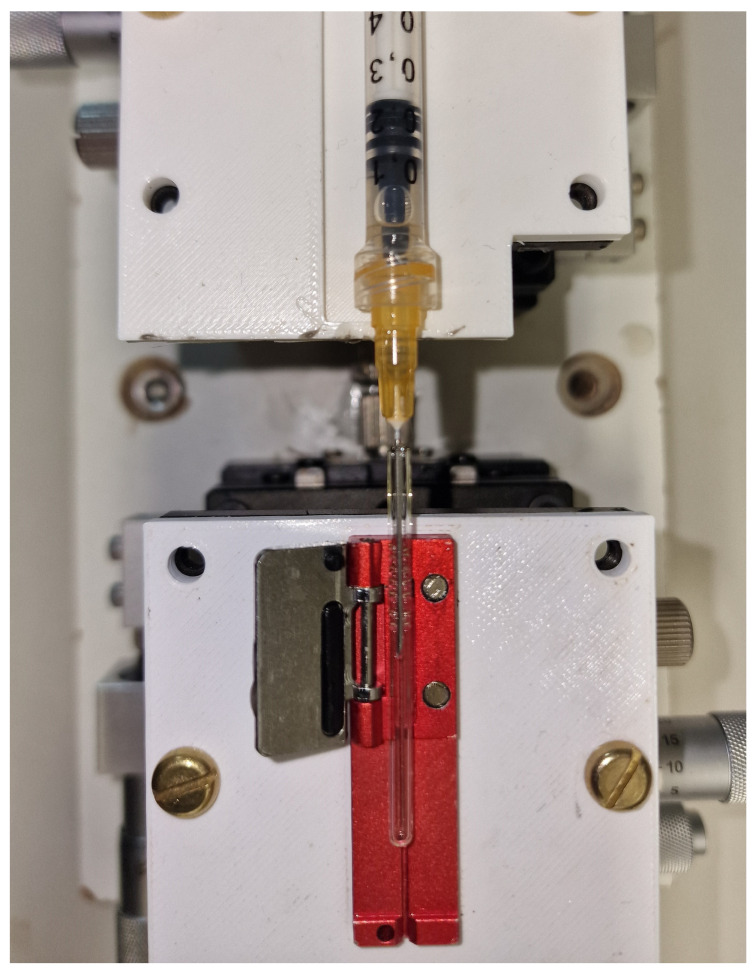
Vertical filling of the melting point capillary with Krytox GPL 105 oil.

The EFPI sensor to be encapsulated was manufactured using the same techniques utilised in Weir et al. [21]. The sensing diaphragm and surrounding outer capillary assembly was encapsulated in a hermetically sealed rigid housing. The housing consisted of a melting point capillary of internal diameter 1.8 mm, outer diameter 2.6 mm and length 50 mm [27]. The melting point capillary and a micro syringe were placed in two custom mounts affixed to two linear stages vertically stacked on a platform. The melting point capillary was placed in the lower linear stage whilst the “oil filled micro syringe” was placed in the upper stage. The needle was then mated with the inner capillary at which point the oil was transfused into the melting point capillary as shown in Figure 2. This was left for 6 h to facilitate any air migration out of the melting point capillary.

**Figure 3:**
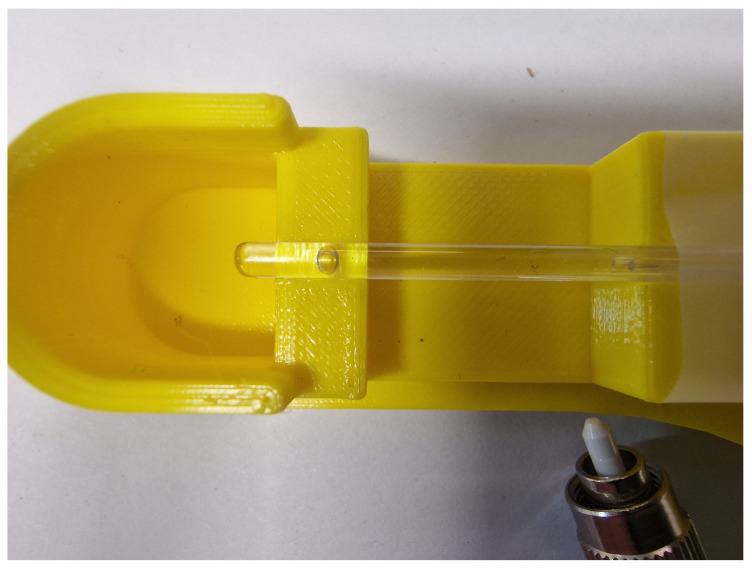
Introduction of air into the hermetically sealed housing via micro-syringe.

The syringe was then slowly removed from the capillary whilst ensuring that sufficient oil was discharged into the capillary to compensate for the volume of the needle in the capillary prior to its withdrawal. The EFPI sensor was then placed into the upper linear stage which previously held the syringe and is aligned coaxially with the fluid-filled capillary. The platform was then rotated from its vertical position to a near horizontal position, with the fluid-filled capillary remaining inclined above the EFPI sensor. The angle was sufficiently low where the viscosity of the oil prohibits any migration from the capillary. Air was introduced to the capillary via a micro syringe to provide sufficient damping as shown in Figure 3.

**Figure 4:**
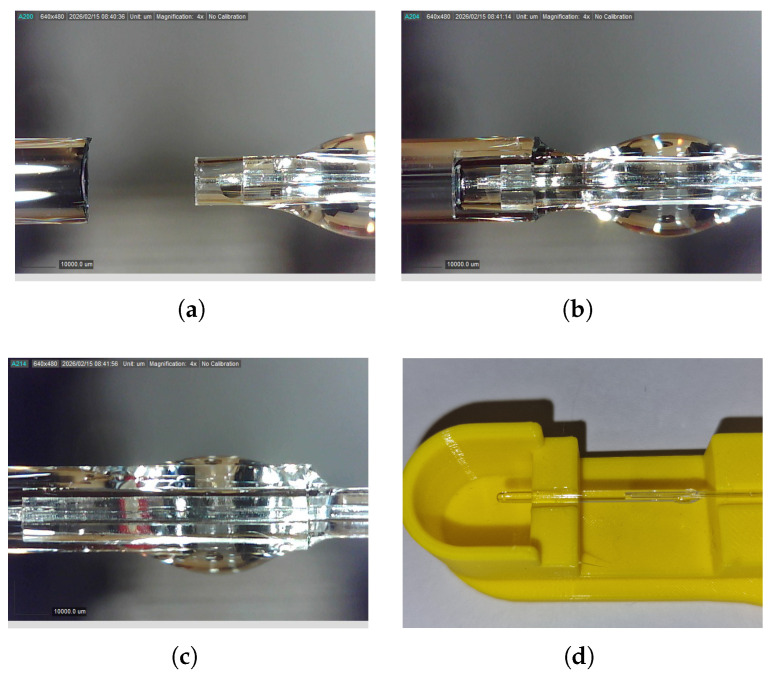
(**a**) Melting point capillary with sensor being introduced, (**b**) initial mating of sensor with melting point capillary, (**c**) fully mated assembly, (**d**) assembled temperature sensor.

Adhesive was applied to the outer sleeve of the sensor and the components were mated. The adhesive was then cured with UV light as shown in Figure 4.

### 3.3. Experimental Setup

#### 3.3.1. Overview

Three sensors were successfully tested with diaphragm thicknesses of 49 μm, 40 μm, and 28 μm. The 49 μm was filled with oil to 80% capacity whilst the 40 μm and 28 μm sensors were filled to approximately 99% oil by volume. This was to maximise the achievable sensitivity whilst maintaining a useable temperature range. A theoretical worked example of the sensitivity of an 80% filled housing with a diaphragm thickness of 49 μm predicts a sensitivity of 0.427 nm/K as demonstrated in Section 4.1.

The 49 μm diaphragm sensor with its hermetically sealed housing was 80% filled with a mixture of Krytox GPL 105 Oil [22] and air. It was then tested in the local lab environment in a large thermal tank where it was exposed to incrementally higher temperatures as it was raised through the water column approximately 16.71 to 20.90 °C, where it demonstrated a sensitivity of 0.405 nm/K. It was subsequently tested in the NSAI [28] where it was tested within a Triple Point of Water cell (TPW) at 0.01 °C [29] and a High Precision Bath (HPB) [30] of approximately 2 °C. The sensor was subsequently tested within the Triple Point of Water cell (TPW) and a Gallium Fixed Point Cell [31] at 29.7646 °C, where it demonstrated sensitivities of 0.255 to 0.32 nm/K.

Two additional sensors with increased ratios of approximately 99% of Krytox/air mixture in the hermetically sealed housings were tested in a benchtop oven [32] from 27 to 35 °C where they demonstrated sensitivities of 21 to 190 nm/K. The spectral responses are presented in the Section 4.

#### 3.3.2. Optical Interrogation

**Figure 5:**
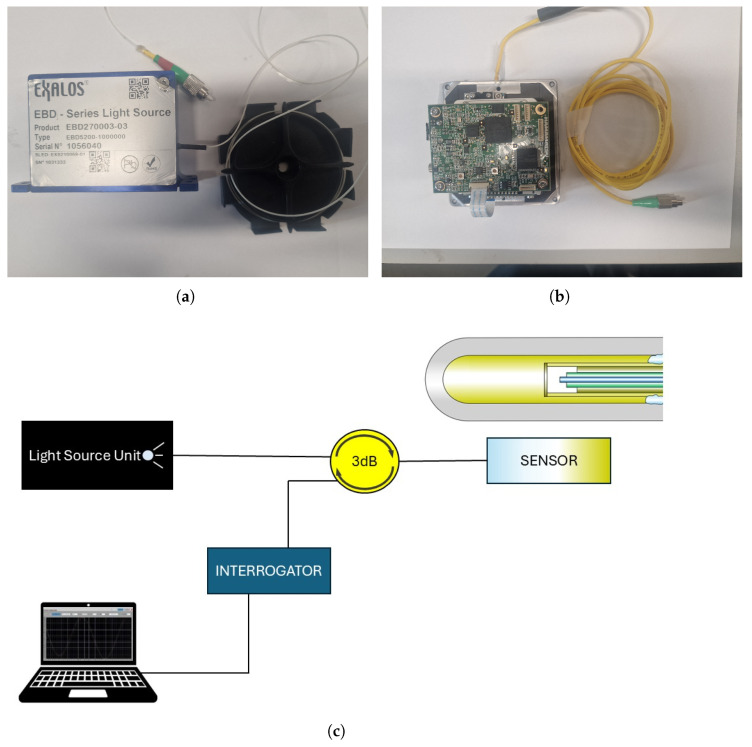
(**a**) Light Source, (**b**) Spectrum Analyser, (**c**) Block Diagram.

A broadband light (BBL) EXALOS EXS210069-01 light source unit generating a Gaussian light output with a half bandwidth of 45 nm centred around 1550 nm is utilised (Ref. [33] as shown in Figure 5a). It propagates through a 3 dB coupler and through to the sensor. The modulated signal is propagated through the SMF and through the 3 dB coupler to the Optical Spectrum analyser (OSA) Figure 5b [34] with a compiled interrogation block diagram shown in Figure 5c. The OSA utilises a LINEAS GaAs image sensing array consisting of 512 pixels, resulting in a wavelength resolution of 0.5 nm over the range of 1510 to 1595 nm. The signal is processed using the LabVIEW^TM^ application as shown in Figure 5c.

Demodulation is performed using Q-point tracking [35]. The sensor is operated at the quadrature point of the interference fringe, defined as the midpoint between a fringe peak and trough where the intensity–displacement slope is maximised and the response is most linear. The effective Q-point is selected on the highest fringe of the spectrum based on the best signal-to-noise ratio. Operation at the Q-point yields the lowest signal distortion and highest sensitivity [35].

In practice, the LabVIEW^TM^ application monitors the intensity at the selected Q-point wavelength and tracks the resulting intensity change as the cavity length varies with temperature. The interrogation window of the Ibsen I-MON 512E spans 85 nm (1510–1595 nm), which defines the free spectral range (FSR) available for demodulation. At the revised sensitivity of 190 nm/K and an operating range of 1.95 K, the maximum total spectral shift is approximately 370 nm. The operating range is governed by the diaphragm fracture pressure rather than the interrogation window; within the 1.95 K range the Q-point remains within the linear region of the fringe and fringe ambiguity does not arise. Phase unwrapping is not required for Sensor A within its operating range. For Sensor C (0.45 nm/K, 166.6 K range), the total spectral shift of approximately 75 nm remains well within the 85 nm interrogation window, and Q-point tracking operates without ambiguity throughout the full operating range.

### 3.4. Experimental Setup, University of Limerick Wet Lab

**Figure 6:**
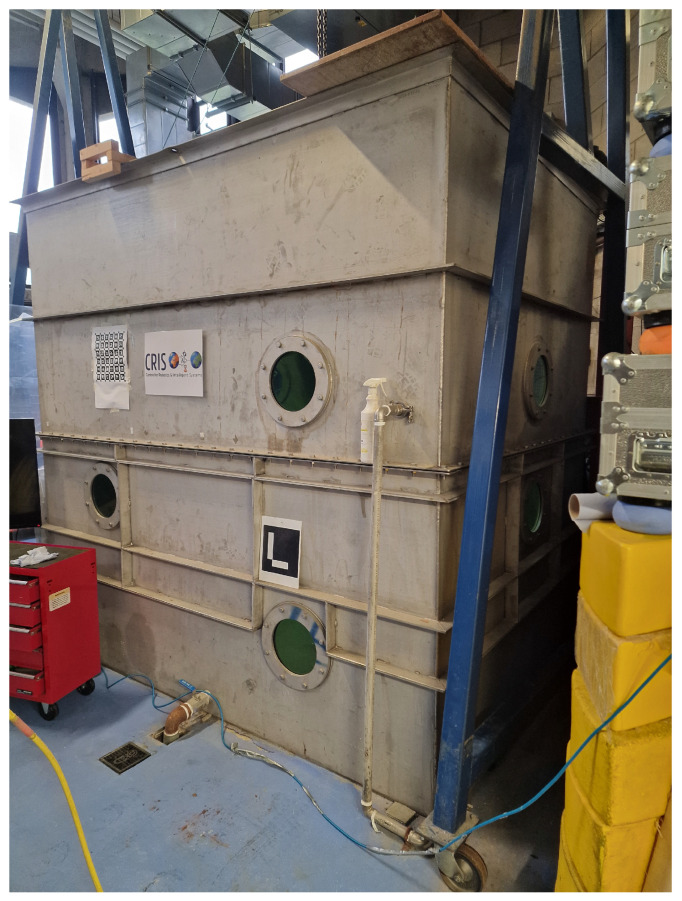
13,000 L tank.

To maximise measurement accuracy under controlled laboratory conditions, experiments were conducted in a 13,000 L tank, as shown in Figure 6, providing a thermally quiescent medium conducive to high-precision sensitivity characterisation.

**Figure 7:**
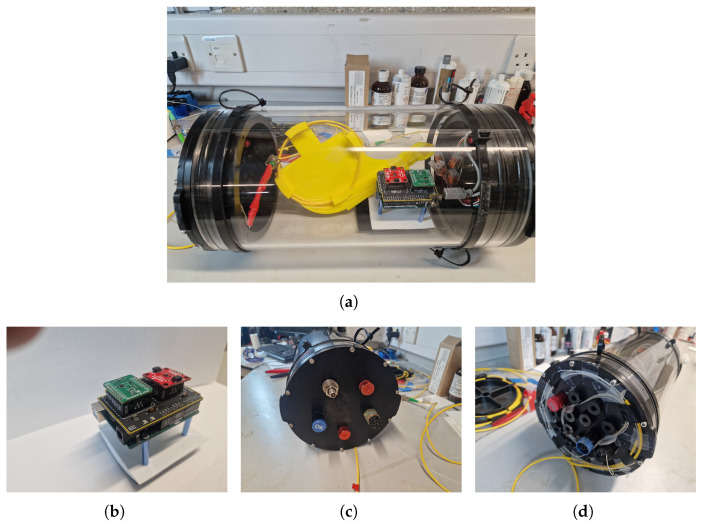
(**a**) Testing Chamber Housing Populated, (**b**) Electronic Temperature Sensor, (**c**) Elec SR breakout, (**d**) Optical Penetrator.

The Sensor was evaluated utilising custom made Hermetically sealed housing as shown in Figure 7a. Integrated into the housing was a high-accuracy, low power Temperature sensor, the TDK InvenSense ICP-10111 [36] integrated onto a Mikroe 4 click board [37], and interfaced with an Arduino UNO [38] as shown in Figure 7b. The Power and Ethernet telemetry for the electronic sensor was transmitted through a subsea bulkhead housing consisting of a Flange [39] and an end cap [40] via a SubConn micro-circular bulkhead connector [41] as shown in Figure 7c. The optical signal was routed through an identical end cap and bulkhead, but was routed through a Blue Robotics Wetlink Penetrator [42] as shown in Figure 7d.

**Figure 8:**
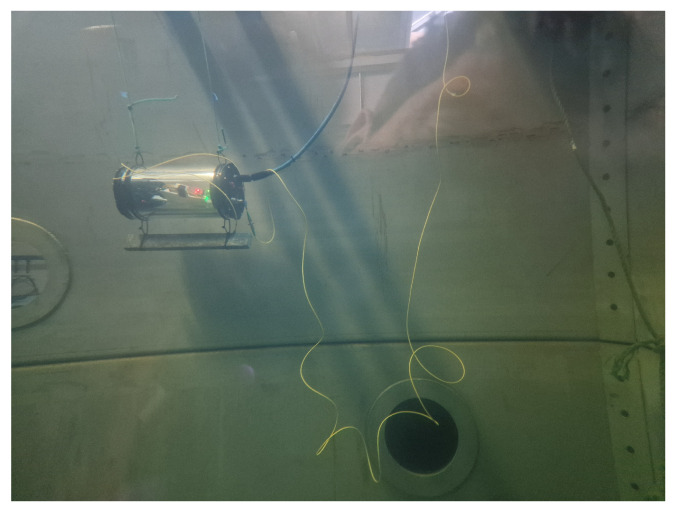
Immersed SR in housing.

Following submersion, the housing was held in suspension within the tank and afforded sufficient time to reach thermal equilibrium with the surrounding medium (Water) as shown in Figure 8. The Sensor was then interrogated and was subsequently raised incrementally through the water column, exposing it to a progressively increasing temperature gradient. The results of this characterisation are discussed later in the Section 4.

### 3.5. Experimental Setup, National Metrology Lab (NSAI) National Standards Authority of Ireland

The testing was conducted at (NSAI) National Standards Authority of Ireland, National Metrology Laboratory in Dublin Ireland [28].

The instrumentation utilised included a Standard Platinum Resistance thermometer(SPRT) [43], a 100 Ω reference resistor [44], a refrigerated temperature calibration bath [45], a Fluke 1595 A Super Thermometer [46], a Triple Point of Water (TPW) Cell [29] and a TPW Maintenance bath [47].

**Figure 9:**
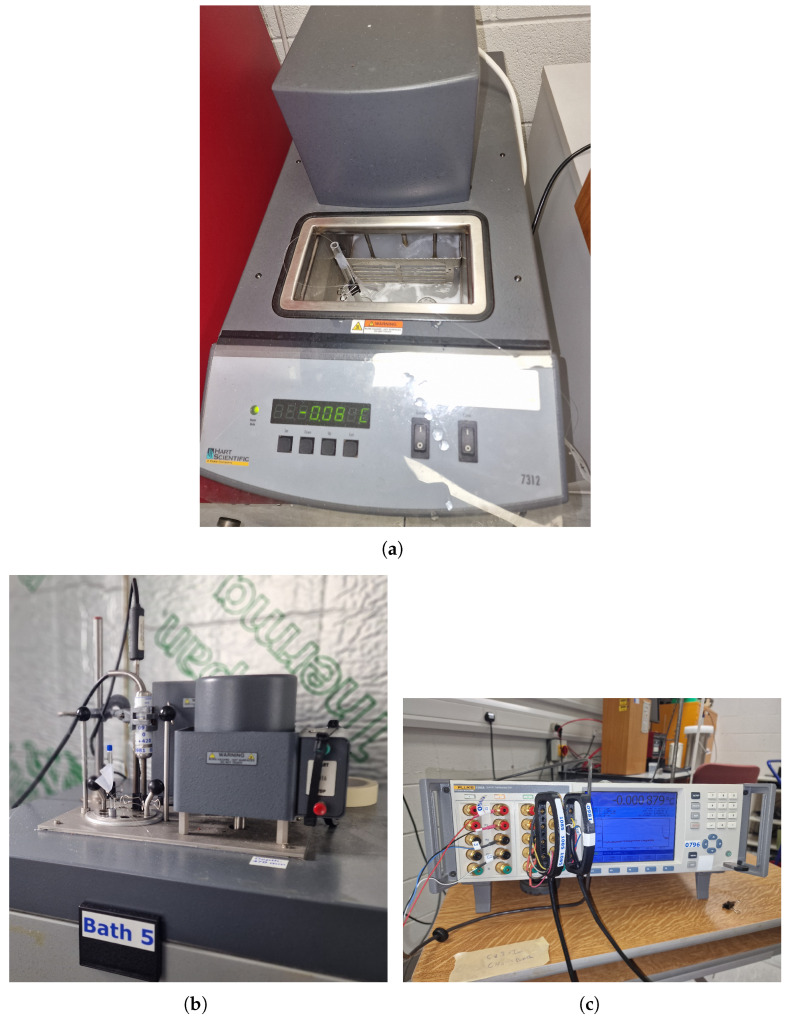
(**a**) TPW Maintenance bath, (**b**) SPRT and Sensor Test Assembly in HPB, (**c**) Fluke Super Thermometer.

The TPW cell is maintained in the TPW Maintenance bath. The TPW maintains a Temperature of 0.01 °C with an uncertainty of +/−0.00033 °C at k = 2 [29] and has traceability in its own right, without the need for a reference SPRT [48]. The sensor is housed in a melting point tube, which is filled with ice water. It was then placed on a melting point ice filled Dewar (Sensor test assembly). After sufficient cooling, the sensor and associated water-filled melting point tube (Sensor Test Assembly) are placed into the TPW housed in the TPW maintenance bath, as shown in Figure 9a, where its response is analysed. The Sensor test assembly was then transferred to the High Precision Bath (HPB) as shown in Figure 9b, where its temperature is referenced with a SPRT [43] interfaced with Fluke Super Thermometer [46] as Shown in Figure 9c and a Tinsley 100 Ω resistor [44], which serves as a reference resistance value for the super thermometer. It then measures the ratio between the reference resistance and the SPRT resistance to determine the exact resistance value of the SPRT [43]. The resultant value is then converted to temperature.

**Figure 10:**
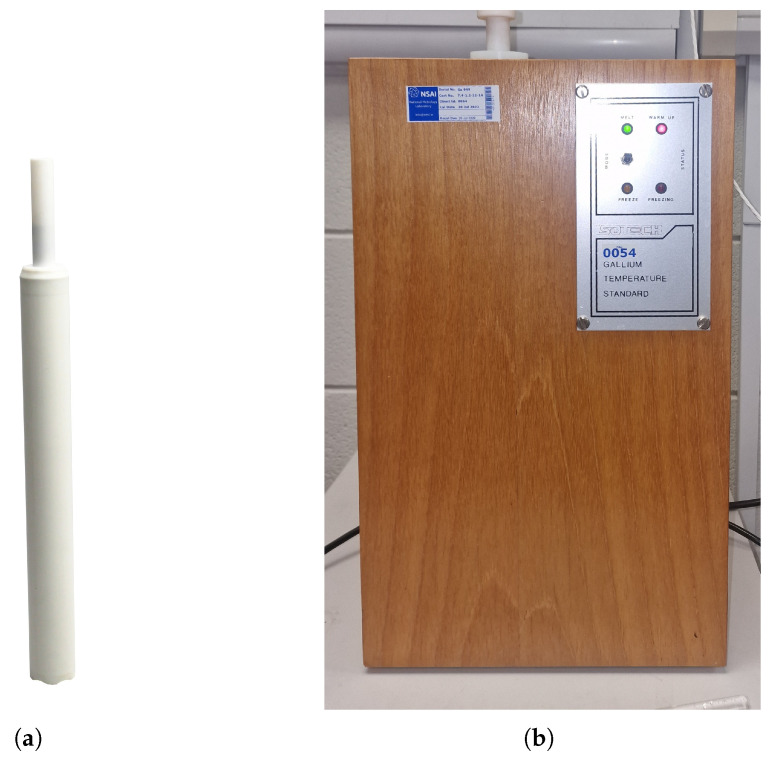
(**a**) Gallium-Fixed-Point-Cell, (**b**) Gallium temperature standard automated control unit.

The Sensor was then transferred to a warming Dewar at approximately 30 °C prior to being inserted into a Gallium Fixed Point Cell [31] at 29.7646 °C with an uncertainty value of +/−0.00075 °C as shown in Figure 10a. This has traceability in its own right, without the need for a reference SPRT [48]. This is maintained in a Gallium temperature standard, automated control Isotech model 17402A [49] as shown in Figure 10b.

The temperature was maintained at 2 °C. The HPB circulators are powered down for a short period of time to reduce the noise. The sensor was then placed back into the TPW cell and monitored.

**Figure 11:**
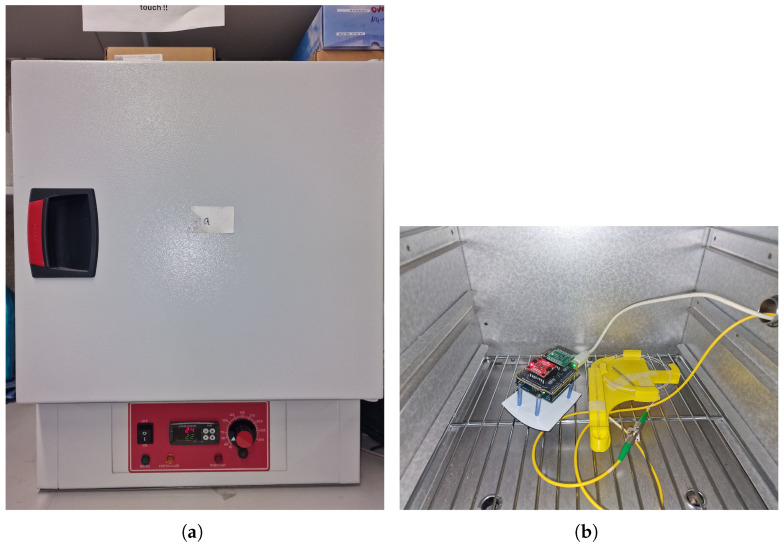
(**a**) Overview of bench oven, (**b**) sensors in bench oven.

Two additional Sensors with diaphragm thickness of 40 μm and 28 μm were filled to over 99% capacity of oil/air ratios in the hermetically sealed housings and were tested in a bench oven [32] as shown in Figure 11a.

These high-sensitivity sensors (Sensors A and B, diaphragm thicknesses 28 μm and 40 μm respectively) were not submitted for NSAI NML testing for two independent physical reasons. First, both sensors were hermetically sealed at approximately laboratory ambient temperature (∼30 °C, close to skin temperature during handling), placing their narrow operating windows of 1.95 K and 5.11 K respectively in the vicinity of the sealing temperature. The NSAI NML test protocol spans from the triple point of water (0.01 °C) to the Gallium Fixed Point (29.7646 °C) with a range of approximately 29.75 K which far exceeds the operating range of either sensor, and exposing them to temperatures near 0.01 °C would displace the operating window entirely outside the design range and risk diaphragm fracture. By contrast, Sensor C has an operating range of 166.6 K which comfortably encompasses the full NML test span, and was sealed at a temperature sufficiently below the Gallium Fixed Point to place the entire NML test range within its operating window. Second, in the vertical NML test configuration the residual air fraction migrates upward under buoyancy toward the diaphragm face. For Sensors A and B, with air fractions of only 0.28% and 0.64% respectively, the resulting bubble is insufficiently large to encompass the entire diaphragm face, producing an inhomogeneous refractive index boundary condition of the partially oil/glass and partially air/glass, which would alter the EFPI spectral response and invalidate the calibration. For Sensor C, with a 20% air fraction, the air volume is sufficient to encompass the entire diaphragm face uniformly in this orientation, maintaining a consistent air/glass boundary condition throughout and ensuring a valid spectral response. Metrological validation of Sensors A and B therefore requires a dedicated test protocol with a narrow temperature window centred on the sealing temperature and a horizontal or inverted sensor orientation, which is identified as a priority for future work.

The temperature was referenced with a high-accuracy, low power Temperature sensor, the TDK InvenSense ICP-10111 [36] integrated onto a Mikroe 4. click board [37], and interfaced with an Arduino UNO [38], as shown in Figure 11b.

## 4. Results and Discussion

**Figure 12:**
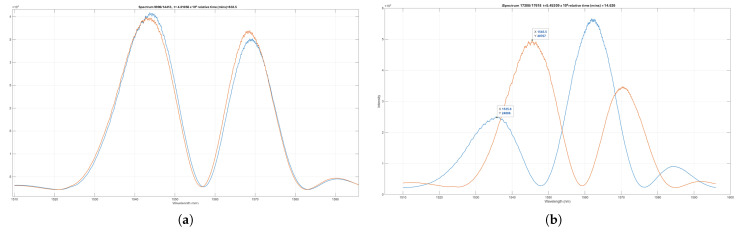
(**a**) Ice Bath to Triple point of Water, (**b**) Triple point of Water to Gallium Fixed Point.

The 49 micron thick diaphragm sensor with the 80% oil fill ratio displayed sensitivities ranging from 0.255, and 0.32 nm/K for the tests conducted in the National metrology Lab in the High precision (HPB) bath at 2 °C to the Triple point of water at 0.01 °C and for the triple point of water at 0.01 °C to Gallium melting point at 29.7646 °C as shown in Figure 12a,b, Where the blue lines represents the spectrum at the initial temperature and the orange line represents the spectrum at the final temperature.

**Figure 13:**
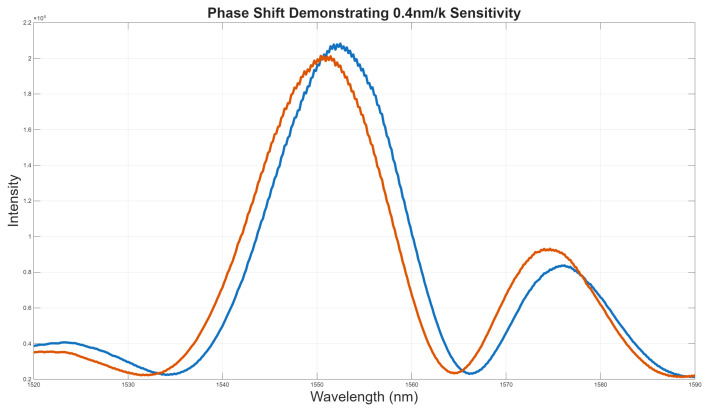
Spectral phase shift for Sensor C (49 μm diaphragm), demonstrating a sensitivity of 0.4275 nm/K, where the blue plot represents the captured spectrum and the orange plot represents the changing spectrum for the change in temperature.

It then demonstrated a sensitivity of 0.405 nm/K for the test conducted in the 13,000 L tank, as shown in Figure 13. The theoretical sensitivity calculated for this sensor shows a sensitivity of 0.4275 nm/K at 80% fill as shown in Section 4.1.

**Figure 14:**
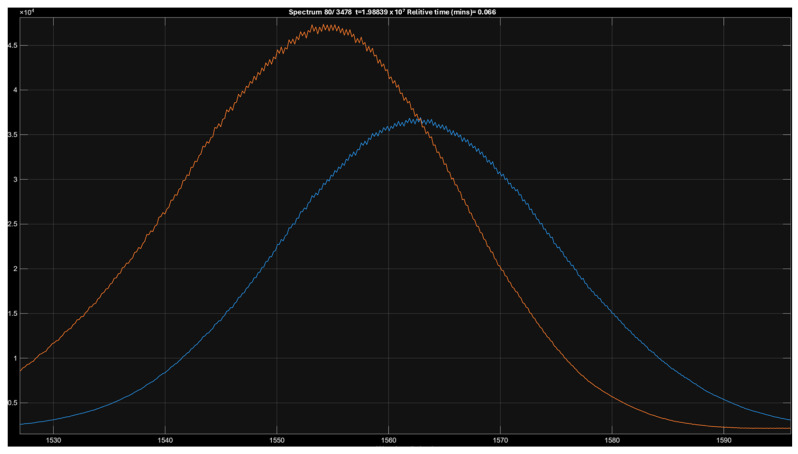
Spectral phase shift for Sensor A (28 μm diaphragm), demonstrating a sensitivity of 190 nm/K, where the blue plot represents the captured spectrum and the orange plot represents the changing spectrum for the change in temperature.

**Figure 15:**
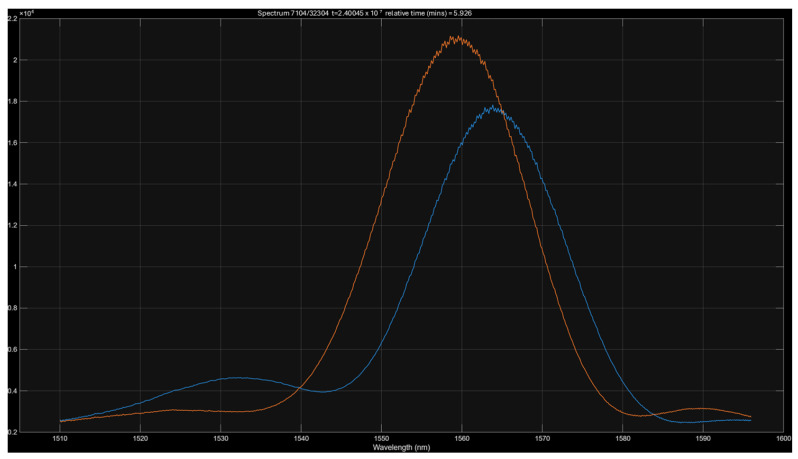
Spectral phase shift for Sensor B (40 μm diaphragm), demonstrating a sensitivity of 21 nm/K, where the blue plot is the spectral response at the initial temperature and the orange plot is the spectral response at the final temperature

The 28 and 40 micron thick diaphragm sensors with the ≥99% oil fill ratio displayed sensitivities at 21 to 190 nm/K, with spectral responses presented in the Section 4, as shown in videos https://youtu.be/l-zoYq_nI1Q (accessed on 14 April 2026) ans as shown in Figure 14 and Figure 15 with a inferred fill fraction of 99.72% and the 40 μm sensor having an inferred fill fraction of 99.36%. The theoretical sensitivity calculated for said sensors shows sensitivities aligning to said values as shown in Section Extraction of Effective Fill Fraction from Measured Sensitivity, 28 and 40 µm Diaphragm Sensors.

**Figure 16:**
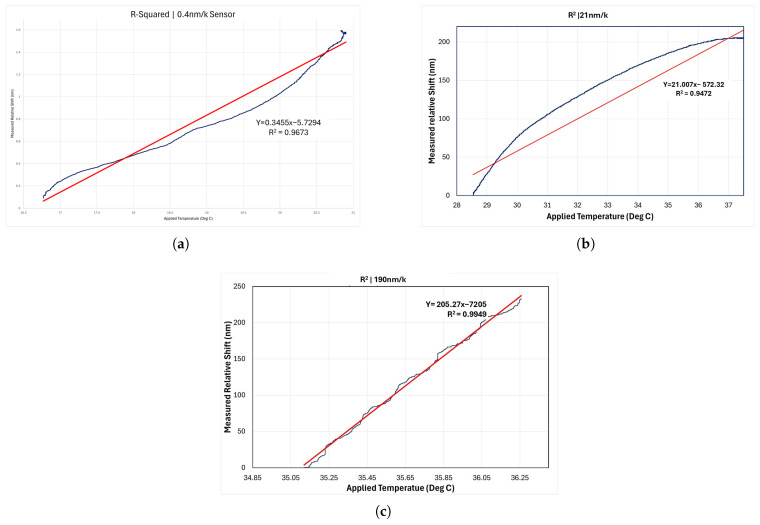
Coefficient of determination (R^2^) for (**a**) Sensor C (0.405 nm/K), (**b**) Sensor B (21 nm/K), and (**c**) Sensor A (190 nm/K) where the blue line represents the measured EFPI optical sensor output as a function of increasing temperature, and the red line represents the linear regression fit to the experimental data.

**Figure 17:**
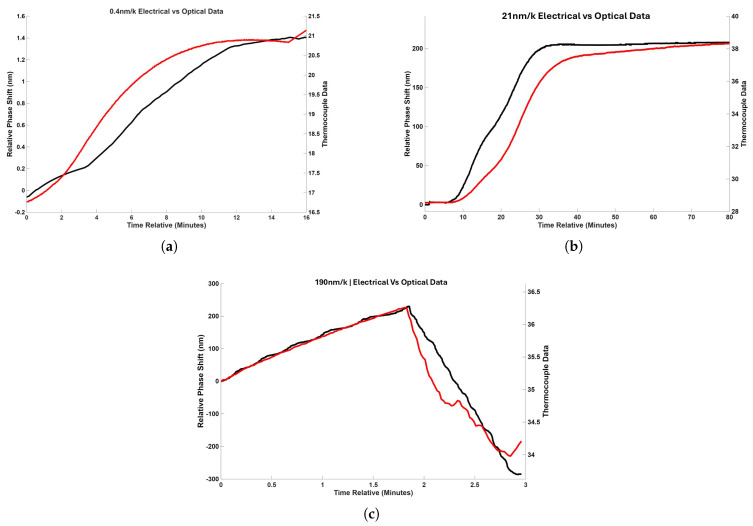
Optical and electronic reference sensor responses for (**a**) Sensor C (0.405 nm/K), (**b**) Sensor B (21 nm/K), and (**c**) Sensor A (190 nm/K) where the black line represents the optical response and the red line represents the thermocouples response.

Furthermore, each of the sensors displayed a linear response as shown in Figure 16, with $R^{2}$ values of 0.943 to 0.995. The complete experimental datasets are publicly available [50]. The response of these sensors is also demonstrated against the thermocouple temperature sensor, the TDK InvenSense ICP-10111 [36] in Figure 17.

### 4.1. Theoretical Sensitivity, 49 µm Diaphragm at 80% Fill

For a fused silica diaphragm of radius a=400 µm, thickness h=49 µm, Young’s modulus E=73 GPa and Poisson’s ratio ν=0.17, the mechanical sensitivity is as defined in Equation (6):

(23) $$S=\frac{3\left(1-\nu^{2}\right)\;\cdot \;a^{4}}{16\;\cdot \;E\;\cdot \;h^{3}}=\frac{3\left(1-0.17^{2}\right){\left(400\times 10^{-6}\right)}^{4}}{16\times 73\times 10^{9}\times {\left(49\times 10^{-6}\right)}^{3}}=5.43\times 10^{-4}\;\mathrm{nm}\;{\mathrm{Pa}}^{-1}$$

The diaphragm deflects $5.43\times 10^{-4}$ nm for every Pascal of applied pressure. At ϕ=0.80 the linearised approximation is valid since $\alpha_{f}\phi \Delta T=0.000872\ll \left(1-\phi \right)=0.20$. The gas and fluid contributions are therefore evaluated using Equation (22). The gas term is:

(24) $$\frac{P_{0}}{T_{0}}=\frac{101325}{293}=345.8\;\mathrm{Pa}\;K^{-1}$$

The fluid displacement term is:

(25) $$P_{0}\;\cdot \;\frac{\alpha_{f}\;\phi }{1-\phi }=101325\times \frac{1.09\times 10^{-3}\times 0.80}{1-0.80}=441.8\;\mathrm{Pa}\;K^{-1}$$

The total composite thermal pressure per kelvin is:

(26) $$\frac{\delta (\Delta P_{\mathrm{total}})}{\delta T}=P_{0}\left(\frac{1}{T_{0}}+\frac{\alpha_{f}\;\phi }{1-\phi }\right)=345.8+441.8=787.6\;\mathrm{Pa}\;K^{-1}$$

The resulting deflection sensitivity is:

(27) $$\frac{\delta y_{\max}}{\delta T}=S\;\cdot \;\frac{\delta (\Delta P_{\mathrm{total}})}{\delta T}=5.43\times 10^{-4}\;\mathrm{nm}\;{\mathrm{Pa}}^{-1}\times 787.6\;\mathrm{Pa}\;K^{-1}=0.4275\;\mathrm{nm}\;K^{-1}$$

A Krytox GPL-105 fill fraction of ϕ=0.80 therefore yields a deflection sensitivity of 0.4275 nm K^−1^ for a 49 µm diaphragm.

#### Extraction of Effective Fill Fraction from Measured Sensitivity, 28 and 40 µm Diaphragm Sensors

The composite thermal pressure model provides a means of inferring the effective oil fill fraction ϕ directly from a measured deflection sensitivity. Since fill fractions above approximately 95% cannot be determined by direct volumetric measurement, the model is used to infer ϕ from the observable sensor response. At these fill fractions the linearised approximation is not valid and the exact expression (Equation (18)) must be used throughout.

Sensor A, measured sensitivity 190 nm K^−1^, h=28 µm

A deflection sensitivity of 190 nm K^−1^ was measured experimentally. The mechanical sensitivity of the diaphragm is:

(28) $$S=\frac{3\left(1-\nu^{2}\right)\;\cdot \;a^{4}}{16\;\cdot \;E\;\cdot \;h^{3}}=\frac{3\left(1-0.17^{2}\right){\left(400\times 10^{-6}\right)}^{4}}{16\times 73\times 10^{9}\times {\left(28\times 10^{-6}\right)}^{3}}=2.909\times 10^{-3}\;\mathrm{nm}\;{\mathrm{Pa}}^{-1}$$

The diaphragm deflects $2.909\times 10^{-3}$ nm for every Pascal of applied pressure. The thermal pressure per kelvin implied by the measured sensitivity is:

(29) $$\frac{\delta (\Delta P_{\mathrm{total}})}{\delta T}=\frac{\delta y_{\max}/\delta T}{S}=\frac{190\;\mathrm{nm}\;K^{-1}}{2.909\times 10^{-3}\;\mathrm{nm}\;{\mathrm{Pa}}^{-1}}=65,320\;\mathrm{Pa}\;K^{-1}$$

Subtracting the gas baseline $P_{0}/T_{0}=345.8$ Pa K^−1^, the fluid displacement term must account for the remainder:

(30) $$P_{0}\;\cdot \;\frac{\alpha_{f}\phi \Delta T}{\left(1-\phi \right)-\alpha_{f}\phi \Delta T}=65,320-345.8=64,974\;\mathrm{Pa}\;K^{-1}$$

Solving numerically using the exact expression (Equation (18)):

(31) ϕ=0.9972(99.72%oil/0.28%air)

The effective fill fraction inferred from the measured sensitivity is therefore ϕ=0.9972. At this fill fraction $\alpha_{f}\phi \Delta T=0.00109$ is 39.1% of the air fraction (1−ϕ)=0.0028, the linearised model underestimates the pressure response and is not applicable here.

Verification substituting ϕ=0.9972 into Equation (18):

(32) $$\frac{\delta (\Delta P_{\mathrm{total}})}{\delta T}=\frac{P_{0}}{T_{0}}+P_{0}\;\cdot \;\frac{1.09\times 10^{-3}\times 0.9972}{\left(1-0.9972\right)-1.09\times 10^{-3}\times 0.9972}=65,320\;\mathrm{Pa}\;K^{-1}$$

(33) $$\frac{\delta y_{\max}}{\delta T}=2.909\times 10^{-3}\times 65,320=190.0\;\mathrm{nm}\;K^{-1}$$

The operating range of this sensor is limited by the diaphragm fracture pressure $P_{\max}=326.67$ kPa, reached within 1.95 K of the sealing temperature.

Sensor B, measured sensitivity 21 nm K^−1^, h=40 µm

A deflection sensitivity of 21 nm K^−1^ was measured experimentally. The mechanical sensitivity of the diaphragm is:

(34) $$S=\frac{3\left(1-\nu^{2}\right)\;\cdot \;a^{4}}{16\;\cdot \;E\;\cdot \;h^{3}}=\frac{3\left(1-0.17^{2}\right){\left(400\times 10^{-6}\right)}^{4}}{16\times 73\times 10^{9}\times {\left(40\times 10^{-6}\right)}^{3}}=9.977\times 10^{-4}\;\mathrm{nm}\;{\mathrm{Pa}}^{-1}$$

The diaphragm deflects $9.977\times 10^{-4}$ nm for every Pascal of applied pressure. The thermal pressure per kelvin implied by the measured sensitivity is:

(35) $$\frac{\delta (\Delta P_{\mathrm{total}})}{\delta T}=\frac{21\;\mathrm{nm}\;K^{-1}}{9.977\times 10^{-4}\;\mathrm{nm}\;{\mathrm{Pa}}^{-1}}=21,048\;\mathrm{Pa}\;K^{-1}$$

Subtracting the gas baseline $P_{0}/T_{0}=345.8$ Pa K^−1^, the fluid displacement term must account for the remainder:

(36) $$P_{0}\;\cdot \;\frac{\alpha_{f}\phi \Delta T}{\left(1-\phi \right)-\alpha_{f}\phi \Delta T}=21,048-345.8=20,702\;\mathrm{Pa}\;K^{-1}$$

Solving numerically using the exact expression (Equation (18)):

(37) ϕ=0.9936(99.36%oil/0.64%air)

At this fill fraction $\alpha_{f}\phi \Delta T=0.00108$ is 17.0% of the air fraction (1−ϕ)=0.0064, the linearised approximation underestimates the pressure response and the exact expression must be used throughout.

**Verification** substituting ϕ=0.9936 into Equation (18):

(38) $$\frac{\delta (\Delta P_{\mathrm{total}})}{\delta T}=\frac{P_{0}}{T_{0}}+P_{0}\;\cdot \;\frac{1.09\times 10^{-3}\times 0.9936}{\left(1-0.9936\right)-1.09\times 10^{-3}\times 0.9936}=21,048\;\mathrm{Pa}\;K^{-1}$$

(39) $$\frac{\delta y_{\max}}{\delta T}=9.977\times 10^{-4}\times 21,048=21.0\;\mathrm{nm}\;K^{-1}✓$$

The operating range of this sensor is limited by the diaphragm fracture pressure $P_{\max}=666.67$ kPa, reached within 5.11 K of the sealing temperature.

## 5. Responses and Limitations of Partially vs. Fully Filled Fluid Housings

As shown in Section 3.1.2, the sensor response is dampened significantly with the introduction of air into the thermal housing, extending the operating temperature range. This relationship is a trade-off between range and sensitivity; sensors must therefore be tuned to specific applications by adjustment of the air/fluid ratio within the housing.

In the case of the 28 µm diaphragm sensor which displayed a sensitivity of 190 nm/K with an inferred fill fraction of 99.72%, the air damping extended its working range from 0.29 K to 1.95 K. Below, as shown in Section 5.1, is a theoretical calculation of the working range of said sensor without air damping. Furthermore, Table 4 compares both the range and sensitivity of said sensor with oil fill volumes from 80% to 99.72%.

### 5.1. Step 1 Diaphragm Mechanical Sensitivity

For a clamped circular diaphragm under uniform pressure, the centre deflection sensitivity is given by Equation (3):

(40) $$S=\frac{3\left(1-\nu^{2}\right)\;\cdot \;a^{4}}{16\;\cdot \;E\;\cdot \;h^{3}}=\frac{3\left(1-0.17^{2}\right){\left(400\times 10^{-6}\right)}^{4}}{16\times 73\times 10^{9}\times {\left(28\times 10^{-6}\right)}^{3}}=2.909\times 10^{-3}\;\mathrm{nm}\;{\mathrm{Pa}}^{-1}$$

The diaphragm therefore deflects $2.909\times 10^{-3}$ nm for every Pascal of applied pressure.

### 5.2. Step 2, Maximum Pressure Before Fracture

The maximum stress in a clamped circular diaphragm under uniform pressure *q* occurs at the clamped edge and is given by [24]:

(41) $$\sigma_{\max}=\frac{0.75\;\cdot \;q\;\cdot \;a^{2}}{h^{2}}$$

Setting $\sigma_{\max}=\sigma_{f}$ and solving for the maximum pressure $P_{\max}$:

(42) $$P_{\max}=\frac{\sigma_{f}\;\cdot \;h^{2}}{0.75\;\cdot \;a^{2}}=\frac{50\times 10^{6}\times {\left(28\times 10^{-6}\right)}^{2}}{0.75\times {\left(400\times 10^{-6}\right)}^{2}}=326.7\;\mathrm{kPa}$$

### 5.3. Step 3, Thermal Pressure for Fully Oil-Filled Cavity

For a rigid cavity completely filled with Krytox GPL 105, the thermal pressure generated per kelvin is governed by the fluid’s bulk thermal pressure coefficient:

(43) $$\frac{\delta (\Delta P)}{\delta T}=\frac{\alpha_{f}}{\kappa_{T}}=\frac{1.09\times 10^{-3}}{9.7\times 10^{-10}}=1,123,711\;\mathrm{Pa}\;K^{-1}=1.124\;\mathrm{MPa}\;K^{-1}$$

### 5.4. Step 4, Temperature Rise to Fracture

The temperature rise δT required to generate $P_{\max}$ in the fully oil-filled cavity is:

(44) $$\delta T=\frac{P_{\max}}{\delta (\Delta P)/\delta T}=\frac{326,700}{1,123,711}=0.291\;K$$

The diaphragm therefore fractures less than 0.3 K above the sealing temperature when fully filled, compared to 1.95 K with the air-damped design at ϕ=99.72%.

## 6. Conclusions and Further Development

This paper has presented a proof-of-concept investigation into a novel hermetically sealed tunable-medium EFPI temperature sensor architecture. A continuously tuneable sensitivity design space spanning 0.45 to 190 nm/K has been demonstrated through variation of the Krytox GPL 105 oil/air fill fraction. The low-sensitivity configuration (Sensor C, 0.45 nm/K) was metrologically validated at NSAI NML against ITS-90 fixed points, whilst the high-sensitivity configurations (Sensor A, 190 nm/K; Sensor B, 21 nm/K) were bench-characterised as preliminary demonstrations of the upper end of the design space. Theoretical and experimental results are in good agreement, as demonstrated in Section 4.1 and Section Extraction of Effective Fill Fraction from Measured Sensitivity, 28 and 40 µm Diaphragm Sensors. An exact nonlinear thermal pressure model has been derived, replacing the linearised approximation shown to be inapplicable at fill fractions approaching unity. The 190 nm/K sensitivity represents an improvement of approximately 21.7× over the closest directly comparable prior art [10], and to the authors’ Knowledge represents the first peer-reviewed journal publication reporting a quantified sensitivity for a diaphragm-based EFPI temperature sensor employing thermal–pressure–mechanical transduction.

The method used to attach the housing to the sensor requires further development.

**Figure 18:**
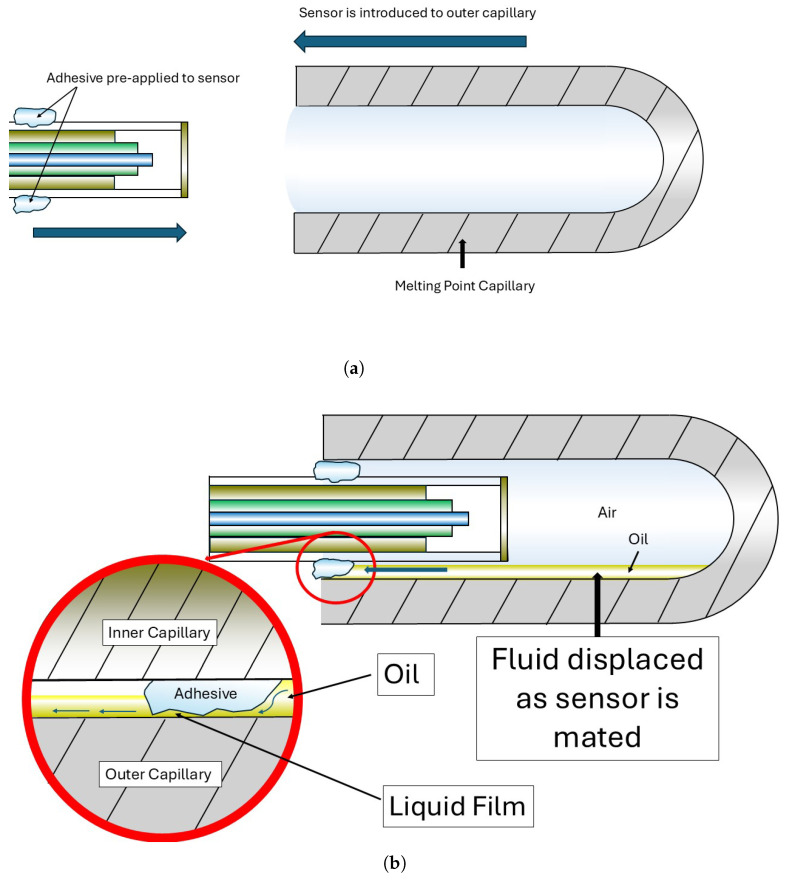
(**a**) Sensor assembly prior to mating; (**b**) sensor assembly during mating, showing thin film fluid displacement.

The current method utilises an adhesive applied to the outer body of the sensor, where the hermetically sealed housing is mated to it via linear stages as shown in Figure 18a. As the adhesive is adhering to a liquid film of the thermal fluid as it is displaced during the mating process, rather than adhering directly to the silica as shown in Figure 18b. This may lead to suspected creep between this fluid housing and the EFPI assembly when the sensor is exposed to large thermal pressures.

The mechanical integrity of the adhesive bond has been assessed as follows. The mechanical integrity of the adhesive bond under thermal pressure loading was assessed for the Safe2Bond 470 IC80 urethane acrylate [51], which forms a cylindrical joint of a 1.5 mm diameter, a 30 mm length and a 0.15 mm thickness between the sensor assembly and the melting point capillary housing. The bond area of $A_{\mathrm{bond}}=2\pi rL=141.4$ mm^2^ is 55.6 times larger than the cavity bore cross-section of 2.54 mm^2^, such that the bore pressure force is distributed over a significantly larger area. The adhesive stress σ at maximum operating pressure $P_{\max}$ is:

(45) $$\sigma =\frac{P_{\max}\;\cdot \;A_{\mathrm{bore}}}{A_{\mathrm{bond}}}$$

At the fracture pressure of each sensor, the adhesive stress reaches a maximum of 18 kPa for Sensor C, 5.9 kPa for Sensor A, and 12 kPa for Sensor B, representing less than 0.09% of the tensile strength of 20 MPa. The resulting bond deformation δ=σt/E at these stress levels is below 6 nm in all cases, producing a cavity volume change of less than 0.001%. Pressure-induced adhesive deformation is therefore negligible and does not contribute to the sensor response.

The outstanding concern with the adhesive interface is not mechanical but chemical: during assembly, the adhesive is applied over a thin film of Krytox oil displaced from the capillary during sensor insertion, as shown in Figure 18b. This results in the adhesive bonding to a fluid film rather than directly to the silica surface, which may reduce bond durability over extended operating periods and under repeated thermal cycling. This is identified as a primary motivation for future development of an all-glass construction in which the capillary housing is fused directly to the sensor assembly, eliminating the adhesive interface entirely.

**Figure 19:**
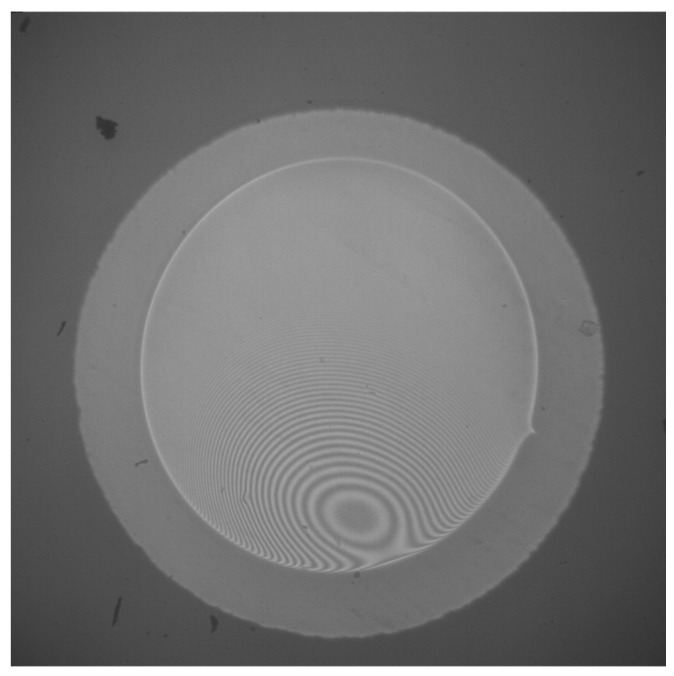
Inner diaphragm geometry.

The diaphragm geometry itself at the post-final reduction stage needs further analysis. It can be seen that when an Arden cleave analyser [52] was used to model the inner face of the diaphragm, as shown in Figure 19, it showed an angle of up to 1.2° [21]. This is attributed to the splice process as excessive heat could facilitate the flow of glass into the capillary. This in itself could lead to a variance of up to 8.4 μm on the inside face alone [21].

The solution to this may lie in the utilisation of a different heating element in the fabrication hardware, i.e., switching from a Graphite to a Tungsten heating element to reach the required temperature in a shorter period of time.

### Future Work

Future development priorities include: metrological validation of Sensors A and B using a dedicated narrow-window test protocol centred on the sealing temperature with a horizontal or inverted sensor orientation; characterisation of hysteresis, repeatability, and thermal cycling behaviour; pressure cross-sensitivity determination for direct-exposure deployments; and progression towards an all-glass hermetically sealed construction in which the capillary housing is fused directly to the sensor assembly.

## Figures and Tables

**Table 1:** Broader contextual overview of fibre optic temperature sensing approaches. Non-diaphragm and gel/expanding-medium sensors are included for context only; no improvement factor is claimed against these devices.

Sensor	Ref	Type	Range (K)	Sensitivity (nm/K)	Diameter (µm)
FBG strain-decoupled, Yan et al.	[17]	FBG	40	0.040	NA
FBG chemical electroplating, Wang et al.	[18]	FBG	95	0.024	NA
FBG, Khan et al.	[19]	FBG	30.5	0.013	NA
PDMS hollow core fibre, Chen et al.	[14]	HCF-FPI	19.3	2.703	NA
Gel-filled capillary EFPI, Zheng et al.	[13]	Gel EFPI	30	0.999–1.174	NA
UV adhesive expanding medium EFPI, Liu et al.	[15]	Gel EFPI	35	4.665	150
UV glue spliced EFPI, Zheng et al.	[13]	Gel EFPI	30	1.174	75
SMF EFPI with integrated FBG (FBG element), Poeggel et al.	[20]	EFPI + FBG	100	0.011	60

**Table 2:** Like-for-like comparison of diaphragm-based EFPI temperature sensors in which the diaphragm is the thermal–pressure–mechanical transduction element. The improvement factor of ∼21.7× is calculated against Poeggel et al. [10], the closest directly comparable prior art. Resolutions for the present work are instrument-limited theoretical values derived from the Ibsen I-MON 512E wavelength fit resolution of <0.5 pm, pending future metrological validation.

Sensor	Ref	Diameter (µm)	Sensitivity (nm/K)	Range (K)	Resolution
PVA diaphragm EFPI, McGuinness et al.	[9]	132	NA	NA	NA
Oil-filled cavity EFPI, Poeggel et al.	[10]	130	8.77	7	NA
This work, Sensor C, (h=49 μm, ϕ=80%)	-	800	0.45	166.6	<1.1 mK
This work, Sensor B, (h=40 μm, ϕ=99.36%)	-	800	21.0	5.11	<24 μK
This work, Sensor A, (h=28 μm, ϕ=99.72%)	-	800	190.0	1.95	<2.6 μK

**Table 3:** Notation and definitions for the diaphragm mechanical characteristics [24].

Symbol	Description	Units
*D*	Flexural rigidity	N · m
*E*	Young’s modulus	Pa
*h*	Diaphragm thickness	m
ν	Poisson’s ratio	dimensionless
*a*	Diaphragm radius	m
*q*	Uniform pressure load	Pa
$y_{\max}$	Maximum deflection at centre	m
$\sigma_{\max}$	Maximum stress at clamped edge	Pa
$\sigma_{f}$	Fracture strength (brittle material)	Pa
*S*	Sensitivity	m/Pa
$P_{\max}$	Maximum pressure before fracture	Pa
$\alpha_{f}$	Volumetric thermal expansion coefficient of fill fluid	K^−1^

**Table 4:** Sensitivity and operating range as a function of Krytox GPL 105 oil fill fraction ϕ for a 28 µm fused silica diaphragm (a=400 µm), computed using the exact thermal pressure model (Equation (18)). The design point at ϕ=99.72% is bold.

Oil (%)	Air (%)	Sensitivity (nm K^−1^)	Range (K)
80.00	20.00	2.297	166.6
85.00	15.00	2.838	119.5
90.00	10.00	3.926	76.2
95.00	5.00	7.239	36.5
97.00	3.00	11.773	21.5
98.00	2.00	17.636	14.2
99.00	1.00	36.657	7.1
**99.72**	**0.28**	**190.000**	**2.0**

## Data Availability

The experimental datasets supporting the results reported in this study are openly available in Zenodo at https://doi.org/10.5281/zenodo.20446927 [50].

## Abbreviations

The following abbreviations are used in this manuscript:

FPI	Fabry–Pérot Interferometer
EFPI	Extrinsic Fabry–Pérot interferometry
HF	Hydrofluoric Acid
ISA	Inner Sensor Assembly
OSA	Outer Sensor Assembly
FSV	Full Scale Value
FSR	free spectral range
MMF	Multimode fibre
SMF	Single Mode fibre
IA	Inner Assembly
OA	Outer Assembly
ID	Inner Diameter
OD	Outer Diameter
RHS	Right Hand Side
LHS	Left Hand Side
UV	Ultra Violet
OFS	OFS Optics
LDC	Large Diameter Cleaver
OSA	Optical Spectrum Analyser
BBL	Broadband Light Source
PC	Personal Computer
SNR	Signal to Noise ratio
SR	Sensor
Pa	Pascal
FBG	Fibre Bragg Grating
MRI	Magnetic Resonance Imaging
UHRTS	ultra-high-resolution temperature sensor
HCF	Hollow Core fibre
PVA	Polyvinyl alcohol
TGA	Thermogravimetric Analyser
NSAI	National Standards Authority of Ireland
VSMOW	Vienna Standard Mean Ocean Water
PDMS	Polydimethylsiloxane
HPB	High Precision Bath
